# Does L2 Proficiency Impact L2-L1 Transfer While Reading L1 Collocations? Evidence From Behavioral and ERP Data

**DOI:** 10.3389/fpsyg.2021.673761

**Published:** 2021-09-29

**Authors:** Agnieszka Otwinowska, Marta Marecka, Alba Casado, Joanna Durlik, Jakub Szewczyk, Marcin Opacki, Zofia Wodniecka

**Affiliations:** ^1^Faculty of Modern Languages, Institute of English Studies, University of Warsaw, Warsaw, Poland; ^2^Faculty of Philosophy, Institute of Psychology, Jagiellonian University, Kraków, Poland; ^3^Department of Psychology, University of Illinois at Urbana-Champaign, Champaign, IL, United States

**Keywords:** L2-L1 transfer, multi-word expression, collocation, ERP, acceptability judgments, cross-linguistic influence

## Abstract

Multi-word expressions (MWEs) are fixed, conventional phrases often used by native speakers of a given language (L1). The type of MWEs investigated in this study were collocations. For bilinguals who have intensive contact with the second language (L2), collocational patterns can be transferred from the L2 to the L1 as a result of cross-linguistic influence (CLI). For example, bilingual migrants can accept collocations from their L2 translated to their L1 as correct. In this study, we asked whether such CLI is possible in native speakers living in the L1 environment and whether it depends on their L2 English proficiency. To this end, we created three lists of expressions in Polish: (1) well-formed Polish verb-noun collocations (e.g., *ma sens* – ^∗^has sense), (2) collocational calques from English (loan translations), where the English verb was replaced by a Polish translation equivalent (e.g., ^∗^*robi sens* – makes sense), and, as a reference (3) absurd verb-noun expression, where the verb did not collocate with the noun (e.g., ^∗^*zjada sens* – ^∗^eats sense). We embedded the three types of collocations in sentences and presented them to L1 Polish participants of varying L2 English proficiency in two experiments. We investigated whether L2 calques would (1) be explicitly judged as non-native in the L1; (2) whether they would evoke differential brain response than native L1 Polish equivalents in the event-related potentials (ERPs). We also explored whether the sensitivity to CLI in calques depended on participants’ level of proficiency in L2 English. The results indicated that native speakers of Polish assessed the calques from English as less acceptable than the correct Polish collocations. Still, there was no difference in online processing of correct and calques collocations as measured by the ERPs. This suggests a dissociation between explicit offline judgments and indices of online language processing. Interestingly, English L2 proficiency did not modulate these effects. The results indicate that the influence of English on Polish is so pervasive that collocational calques from this language are likely to become accepted and used by Poles.

## Introduction

In all languages, certain words co-occur and form fixed sequences called multiword expressions (MWEs, Siyanova-Chanturia, 2013, 2015), such as collocations (e.g., *take a picture*). For learners of second or foreign languages (L2) word choices in L2 MWEs may sometimes be arbitrary (Szudarski and Conklin, 2014), but native speakers of a language (L1) can judge that “something is wrong” when certain words in the MWE are replaced by other words. For example, when native speakers of English are asked to make an explicit judgment on collocations, most would prefer the phrase *take a picture* to the phrase *make a picture*. In a similar context, a native speaker of Polish would prefer *zrobić zdjęcie* (make a picture) to *wziąć zdjęcie* (take a picture, which in Polish implies taking the picture in one’s hands). However, while native speakers might have explicit, metalinguistic opinions about MWE use, are they equally sensitive to such subtle differences as *make/take a picture* when processing collocations?

Evidence suggests that for bilinguals living in an L2 environment, explicit judgments between native and non-native collocations might become blurred (Laufer, 2003; Schmid and Köpke, 2017). This is due to the influence of the L2 on the L1 (Jarvis and Pavlenko, 2008), known as cross-linguistic influence (CLI). In our study, we asked whether the native and non-native distinctions between collocations can become blurred due to CLI in the case of those native speakers who live in the L1 environment and who use English as the “global language” (Seidlhofer, 2013). We presented L1 Polish participants of varying L2 English proficiency with sentences containing Polish collocations, as well as sentences where those collocations were replaced by their calques (loan translations) from English. We investigated whether such calques would (1) be explicitly judged as non-native and (2) evoke differential brain response than their native Polish equivalents in the event-related potentials (ERPs). We also explored whether sensitivity to CLI in calques depends on participants’ level of proficiency in L2 English.

### What Are Multi-Word Expressions and How to Identify Them?

To express a specific meaning, native speakers of any language often use multiword sequences, fixed expressions or phrases characterized by a degree of connectedness and recognized as conventionalized (Siyanova-Chanturia, 2013). Those multiword sequences can be called formulaic expressions and formulaic sequences (Wray and Perkins, 2000; Kecskes, 2015; Carrol and Conklin, 2020), multiword items (Siyanova-Chanturia and Omidian, 2019), or multi-word expressions (MWEs, Siyanova-Chanturia, 2013). MWEs are defined as combinations of words for which syntactic or semantic properties of the expression cannot be obtained from their component parts (Sag et al., 2002). There are many types of MWEs, including grammatical expressions (*is going to*), phrasal verbs (*look up*), situation bound utterances (*How can I help you?*), binomial expressions (*bread and butter*), idioms (*take the bull by the horns*), and collocations (*take a picture*; Wray, 2005; Kecskes, 2015; Siyanova-Chanturia, 2015). Although MWEs differ in terms of their fixedness (how restricted the word combination is) and compositionality (the degree to which the meaning of the MWE stems from the meaning of the separate words), they can be identified based on their recurrence in the given language. This means that in natural language MWEs recur more frequently than comparable phrases that are less fixed (Carrol and Conklin, 2020).

Based on the characteristics of fixedness and recurrence, two predominant methods of identifying MWEs have been proposed. The “phraseological approach” uses native speaker intuitions in the assessment of how fixed or non-compositional particular word combinations are with respect to their meaning (Siyanova-Chanturia, 2015; Siyanova-Chanturia and Omidian, 2019). A more quantitative method based on language corpora, is the “frequency-based approach” (Sinclair, 1991; Durrant and Schmitt, 2009). It investigates the relationship of words that co-occur within texts with greater than random probability. In a language corpus, two or more words are considered to be a MWE if they co-occur more often than predicted from the frequencies of the separate words. For instance, collocations (such as, *take a picture*) are pairs of words that co-occur more frequently than we would expect by chance (Sinclair, 1991; Carrol and Conklin, 2020) and are compositional, so their meaning depends on the meanings of the particular words. However, the words in a collocation will be more strongly associated than words in other word combinations (*take a picture* vs. ^∗^*make a picture*). Thus, statistical metrics of association strength are employed in the frequency-based approach to identifying MWEs. Most notably, they include the t-score, which is the confidence with which we can assert that there is an association between two words, and the mutual information (MI) score^1^, which is the strength of co-occurrence between two words that form an MWE (Sinclair, 1991; Siyanova-Chanturia and Omidian, 2019; Carrol and Conklin, 2020). Because both approaches to identifying MWEs (frequency-based and phraseological, intuition based) have their own advantages and limitations (see Wray, 2005; Durrant and Schmitt, 2009), and are often employed in a complementary fashion (Siyanova-Chanturia and Omidian, 2019), both will also be used in the current study when creating experimental materials.

### MWEs in L1 Use and Processing

Multi-word expressions are commonly used by native speakers, especially in speech, and the frequency of MWEs in a native speaker’s lexicon is almost equivalent to that of single words (Wray and Perkins, 2000). Linguistic literature suggests that native speakers use MWEs mainly for reasons of speech economy, because they are stored in memory as prefabricated “language chunks,” are selected as wholes for production, and do not need to be decomposed to access their meaning in comprehension (Sinclair, 1991; Wray, 2005, 2012). Indeed, native speakers process MWEs such as idioms, binominals, and collocations faster than other word combinations. This manifests itself in faster reaction times in behavioral tasks and reading (e.g., Arnon and Snider, 2010; Vespignani et al., 2010; Rommers et al., 2013; Vilkaité, 2016; Vilkaité and Schmitt, 2019; Carrol and Conklin, 2020). For instance, Carrol and Conklin (2020) used eyetracking to compare reading times of three types of MWE, that is idioms, binomials, and collocations relative to control phrases. The results showed that native speakers of English read all three MWE types embedded in sentences faster than control phrases. Also Vilkaité (2016) found that English native speakers read verb-noun collocations (e.g., *provide information*) embedded in sentences more quickly than control phrases (e.g., *compare information*), even in non-adjacent configurations separated by three other words (e.g., *provide some of the information*). Vilkaité interpreted this as evidence against the suggestions that collocations and other MWEs are stored and processed as wholes and remain unanalyzed (Wray, 2005, 2012). Rather, what these data imply is that parts of a MWE are more predictable than other word combinations occurring in discourse.

The mechanism of prediction underlying discourse comprehension is based on the interplay between the language information already processed by our brain and the information currently being processed (Vespignani et al., 2010). Thanks to the previously processed linguistic information, the memory representation of a given word becomes activated even before this word occurs in the input directed to the person hearing or reading that discourse stretch (Szewczyk and Schriefers, 2018). If the sequence or co-occurrence of some words is highly predictable, the words previously processed activate the words to occur. Some studies into predictive effects in language comprehension have investigated such MWEs as idioms (e.g., Vespignani et al., 2010; Rommers et al., 2013), binominals and collocations (Siyanova-Chanturia et al., 2017; Carrol and Conklin, 2020). Their results point out that, indeed, parts of a MWE are highly predictable, most likely because they are composed of words that often co-occur in fixed patterns. Thus, the activation of one element of an MWE activates the other elements faster than in the case of less fixed phrases, leading to faster processing of the entire expression (Arnon and Snider, 2010).

The predictability of MWEs when processed by native speakers can also be detected in the presence of specific components measured in the scalp-recorded event-related potentials (ERPs) used in the study of language comprehension. One of those components is the N400 (Kutas and Hillyard, 1980), which is a broad negative deflection that begins 200–300 ms after a word has been presented and reaches its peak after approximately 400 ms. The N400 always occurs when a native speaker of a language is perceiving any stimuli that are conceptually meaningful. It reflects the degree to which the conceptual representations associated with the stimulus have already been active in long-term memory. Accessing the representations that are already active leads to less negative N400 amplitudes than accessing stimuli that are less activated in memory. Comprehending a coherent sentence often leads to a pre-activation of meanings that are likely to occur in the upcoming part of a sentence. Thus, words that are congruent with the sentence often elicit N400 components with a less negative amplitude, but reading words incongruent with the prediction gives rise to a large N400 component. In other words, it is a typical finding that the amplitude of the N400 correlates with the extent to which a word’s meaning is congruent with the meaning of the preceding context (Kutas and Federmeier, 2011; Szewczyk and Schriefers, 2018).

Although ERP studies on MWE processing are still limited, they all show that once the reader encounters an MWE and reads the first word, the predictability of the words in the remaining part of the MWE greatly increases. Cases when native speakers process MWEs in comparison with other, non-fixed expressions yield the less negative N400 amplitudes. This has been demonstrated for the processing of idioms in Dutch and Italian (e.g., *cry over spilt milk* vs. *cry over spilt coffee*; Vespignani et al., 2010; Rommers et al., 2013), binominal phrases in English (e.g., *knife and fork* vs. *spoon and fork*; Siyanova-Chanturia et al., 2017) and collocations in Spanish (*quite the opposite* vs. *all the opposite*; Molinaro and Carreiras, 2010). The reduced N400 when processing MWEs relative to the processing of other non-fixed word combinations indicates that the parts of an MWE were highly expected, possibly because their structure and meaning were stored in memory.

Yet another component reported in several ERP studies on MWEs is the P300, a positivity occurring in the 250–350 ms time-window on parietal electrodes (Molinaro and Carreiras, 2010; Vespignani et al., 2010; Siyanova-Chanturia et al., 2017). The P300, which peaks at around 300 ms with an onset at around 250 ms after the stimulus, follows the processing of highly formulaic MWEs and also indexes reactions to predictable and unpredictable stimuli. For instance, the more positive P300s for words within idioms relative to other (literal) word combinations have been interpreted as associated with expectancies that arise during stimulus processing (Vespignani et al., 2010; Siyanova-Chanturia et al., 2017). Vespignani et al. (2010) interpreted the occurrence of the P300 as reflecting the match of the actual input (the idiom fragment) to the stored template (a specific configuration) retrieved from semantic memory. Also Molinaro and Carreiras (2010), who studied the processing of Spanish collocations, interpreted the increased P300 as an index of initially recognizing that the sequence was a collocation, which lead to pre-activating the word completing the collocation. Finally, Siyanova-Chanturia et al. (2017) found the P300 component in the processing of binominals. They attributed the presence of the component to template matching. They explained that the component was detected, because when processing the binomial, participants expected particular unique words to occur, and they did not have to perform a lexical search for the item. The first part of the phrase was simply matched to a known template of this phrase retrieved from memory. The authors argued that the P300 and N400 components represented two different processing stages, one associated with the recognition of a unique, predictable and prefabricated routine (leading to the increased P300 amplitudes) and the other associated with facilitated processing and semantic integration (eliciting the reduced N400 amplitudes).

Overall, we have argued that MWEs (such as idioms, binominals, and collocations), which are characterized by a high degree of connectedness, are ubiquitous in native speaker discourse. MWEs can be identified in two ways in native speech and writing (Siyanova-Chanturia and Omidian, 2019): using native speaker judgments (the phraseological approach), and by applying measures based on word co-occurrence (the frequency-based approach). During comprehension tasks, native speakers process MWEs in their language faster than other less fixed expressions, which is revealed by their faster reaction times to such stimuli. MWE processing elicits specific ERP components. In particular, MWEs (such as idioms, binominals, and collocations) commonly evoke the reduced N400 component, meaning that they are highly predictable to native speakers. The processing of MWEs can also be accompanied by the P300 component, possibly indexing the matching of the input to the stored template. However, as claimed by Siyanova-Chanturia et al. (2017), the ERP research into MWEs is still in its infancy. Also, less is known about the comprehension of MWEs in speakers who are exposed to more than one language and there is a considerable shortage of ERP studies in this area.

### Cross-Linguistic Influences in the Use and Processing of Collocations

Speakers of two languages may not use and process MWEs similarly across their languages. In particular, they do not rely on MWE’s in L2 speech and writing as much as in L1 (Pawley and Syder, 2014). Even advanced non-native L2 users produce fewer MWEs when compared to native speakers. However, some MWEs are over-represented, and others underrepresented in the L2 output (Durrant and Schmitt, 2009; Gozdawa-Gołębiowski and Opacki, 2018). This might depend on the L2 proficiency of the speakers. For instance, less proficient L2 users more eagerly rely on transparent phrasal verbs than on idioms and collocations (Kecskes, 2015), and may use L1 collocational patterns when speaking the L2 (Wray and Perkins, 2000). This suggests that the use of MWEs in the L2 is related to cross-linguistic influence (CLI) between the L1 and L2 of a particular speaker. CLI is defined as “the influence of a person’s knowledge of one language on that persons’ knowledge or use of another language” (Jarvis and Pavlenko, 2008, p. 1).

Despite the linguistic evidence on the limited role of MWEs in L2 use and CLI from the L1, psycholinguistic evidence reveals that L2 users are sensitive to collocational patterns in both their L1 and L2. The processing of L2 collocations is faster than that of other L2 phrases, just like in the case of L1 collocations (Siyanova-Chanturia, 2017). However, the speed of processing L2 collocations depends on several factors. First of all, L2 users react faster to more frequent than to less frequent L2 collocations (Siyanova and Schmitt, 2008; Durrant and Doherty, 2010), which indicates that collocational processing might depend on the amount of exposure to L2 input. Second of all, incongruent L2 collocations (i.e., ones which do not have an equivalent in the L1) are more difficult to process than L2 collocations congruent with L1 patterns (Yamashita and Jiang, 2010; Wolter and Gyllstad, 2011, 2013), and are also more difficult to learn (Szudarski and Conklin, 2014). As proposed, when the L2 user encounters an L2 collocation which is congruent with the L1 collocation, its appearance triggers the activation of the L1 translation equivalent and results in a faster recognition of the L2 collocation (Wolter and Gyllstad, 2011, 2013; Wolter and Yamashita, 2015). All these studies point to some carry over or CLI effects across the speakers’ languages in collocational processing and learning. Clearly, L1 knowledge influences the use and processing of the L2, indicating that both L1 and L2 systems are activated when L2 users comprehend MWEs.

However, assuming that both languages are indeed activated, a valid question about CLI, is to what extent the L2 collocational knowledge influences the knowledge and processing of collocations in the L1. Although the definition of CLI specifies that CLI effects can be bidirectional (see Cook, 2003, Cook, 2016; Jarvis and Pavlenko, 2008), there is surprisingly little evidence that collocational knowledge can be transferred not only from the speaker’s L1 to the L2, but also from the L2 to the L1. Such evidence has been studied mostly in migrant contexts and has been associated with language attrition – changes in the L1 system due to the exposure and use of the L2 (see Schmid and Köpke, 2017 for a discussion). For example, Marian and Kaushanskaya (2007) examined the patterns of lexical use and attrition in narratives produced by Russian migrants to the United States when they spoke English and Russian. The authors found examples of overt use of words borrowed from the language other than the one spoken and examples of CLI in the use of MWEs. Overall, bilinguals transferred more when speaking the L2 and borrowed more when speaking their L1. The only study on the CLI from L2 to L1 in the case of collocations that we are aware of is the one by Laufer (2003), who examined the acceptability of collocations transferred or calqued from Hebrew to Russian in the case of Russian migrants to Israel. In an acceptability judgment task, both Russian monolinguals and Russian-Hebrew bilingual migrants were asked to explicitly assess whether some sentences in Russian were acceptable or not. In Laufer (2003) study, many of the bilingual Russian migrants accepted collocational calques from their L2 Hebrew as correct in their L1 Russian. This was possibly due to the high exposure of Russian speakers to the L2 Hebrew collocational patterns, which resulted in adopting the frequent ones within the L1.

However, Schmid and Köpke (2017) challenge the view that attrition or restructuring of the L1 under the influence of the L2 is a phenomenon specific only to migrant contexts. On the contrary, they claim that the process of CLI from L2 to L1 affects all bilinguals, and not only those who are immersed in using the L2 and make little use of their L1. Still, although there is a growing body of research on MWE processing and use among monolinguals, in the case of bilingual speakers most studies examined MWEs in their L2. Of these, studies of collocations are less numerous than those of other MWEs, and most research, especially such that relies on the use of ERP and eye-tracking, focuses on the processing of idioms (Siyanova-Chanturia, 2013, 2017). To our knowledge, there is nearly no research on the use of collocational patterns by L1 speakers living in the L1 environment who have a relatively high L2 proficiency. Our study aims to fill this gap.

## The Current Study

The goal of the study was to explore the sensitivity of Polish native speakers to CLI from their L2 English in the area of MWE and test whether the sensitivity is modulated by their L2 proficiency. Following Schmid and Köpke (2017), we assumed that due to contact with the L2, collocational patterns can be transferred from the L2 to the L1. We argue that this type of CLI is possible not only in the case of migrants immersed in the L2, but also in the case of L1 speakers. Although they live in the L1 environment, they have some knowledge of and contact with L2 English, a language of high social prestige and wide presence at the societal level (in the media, work environments, and education).

We assumed that as a result of intensive language contact (Winford, 2003) and CLI (Jarvis and Pavlenko, 2008), some MWEs, such as collocations can be borrowed and transferred from English to Polish. Here, we focused on collocational calques (loan translations) from L2 English, where the L2 words are replaced by semantically equivalent L1 Polish words (Haugen, 1950). Calques can be created by direct translation from the L2 to the L1. Polish collocational calques are then composed in accordance with the English pattern, but consist entirely of Polish words. Because calques contain only L1 words, they are less noticeable to Polish native speakers than foreign loanwords (Otwinowska-Kasztelanic, 2000), and can eventually become so frequent that they might get accepted as a part of the L1 language system and begin to be used by the speakers of the L1 according to the L2 pattern (Winford, 2003).

A good example of calques in Polish are novel verb-noun collocations based on English, e.g., ^∗^*wziąć autobus* (“take the bus”), which have penetrated the Polish language and are sometimes used by Poles, even though they are incorrect from a prescriptive standpoint. In the case of ^∗^*wziąć autobus*, a very similar Polish collocation exists, namely *wziąć taksówkę* (“take a taxi”), but it is restricted to only that particular type of transportation. We propose that the mechanism that leads to constructing such novel Polish collocational calques involves replacing the original frequent L1 Polish verb (*pojechać* – “go”) by another frequent L1 verb (*wziąć –* “take”), typical of the English (L2) collocation (^∗^*wziąć autobus* – “take the bus”). This results in creating a meaningful word combination, superficially similar to the original Polish collocation.

In this study, we focused on verb-noun collocational calques from English (^∗^*wziąć autobus*) as compared with correct Polish collocations (*pojechać autobusem*) and with absurd expressions (^∗^*zjeść autobus* – “eat the bus”). We tested whether Polish L1 speakers with L2 English living in a Polish-speaking environment (1) judge the English collocational calques as acceptable, and (2) show sensitivity to the calques on a neural level as demonstrated by the ERPs. Moreover, we explored whether individual variability in L2 proficiency modulates the magnitude of these effects. To this end, we ran a behavioral study aiming to check the acceptability of the correct, calqued, and absurd collocations, and an ERP experiment testing the neural response to correct collocations vs. calques and absurd collocations. We expected that the collocational calques (^∗^*wziąć autobus*) and absurd expressions would be judged by bilingual native speakers of Polish as less acceptable than the correct collocations (*pojechać autobusem*). We also expected that the novel strings of words (calques and absurd expressions) would not be processed by bilingual native speakers of Polish similarly to the existing collocations (see Materials for the mean MI scores of the three groups of expressions and Supplementary Appendix 1 for each MI score).

According to the ERP studies reviewed above, as participants read through a known (correct) collocation, the last word of the collocation should be predictable given the preceding words. It thus should lead to a reduced N400 component (Molinaro and Carreiras, 2010), compared to unknown or incorrect collocations. Also, relative to other non-fixed word combinations (not MWEs), the processing of correct collocations should activate the template matching mechanisms for linguistic information that is uniquely predictable, as revealed by the increased P300 component (Vespignani et al., 2010; Siyanova-Chanturia et al., 2017).

Following Molinaro and Carreiras (2010), we assumed that the effects would be time-locked to the last word of the collocation, which is the noun. In the correct Polish verb-noun collocations, on the presentation of the verb, the noun should become highly predictable and expected for native speakers of Polish. Thus, in our study, the correct Polish collocations (relative to English calques and absurd expressions) should evoke a reduced N400 time-locked to the onset of the noun, indicating facilitated semantic access or integration. The correct collocations should also evoke an increased P300 to the noun, indicating stronger activation of template matching than in the case of the non-fixed expressions (calques and absurd collocations). In contrast, the processing of the absurd collocations should evoke an increased N400 and a decreased P300 component, time-locked to the onset of the noun. The processing of the nouns in the calques should not be similar to those in the correct collocations demonstrating detection of anomaly, unless the L2 calques are already becoming accepted as a part of the L1 language system. We assume that the effects for collocational calques may be modulated by the participants’ L2 English proficiency. More specifically, the higher L2 proficiency, the less sensitivity to the calques should be observed (decreased N400 and increased P300 components).

## Experiment 1 – Behavioral Online Study

### Methods

#### Participants

We recruited 35 native speakers of Polish, mean age 22.38 years (range 19–36; *SD* = 4.17). Participants reported an average of almost 2 h (119 min) of English use daily (*SD* = 2.19). Their self-rated proficiency in English equaled 5.12 (range 3–7; *SD* = 1.11), as measured on a 7-point Likert scale (where 7 meant native-like proficiency). As for the proficiency range, 4 participants reported level 3, 6 participants reported level 4, 11 participants reported level 5, 11 participants reported level 6, and 3 participants reported level 7.

#### Materials

For the study, we created three lists of Polish language multi-word stimuli that consisted of verb-noun combinations. They included well-formed Polish collocations, collocational calques (verb-noun equivalents of well-formed English collocations translated word for word into Polish), and absurd expressions (verb-noun combinations where the verb did not match the noun semantically). To create the stimuli, we performed a frequency based cross-analysis of two national corpora, the British National Corpus (BNC, representing English) and the balanced subsections of the National Corpus of Polish (Narodowy Korpus Języka Polskiego, NKJP, representing Polish). For the BNC, we used the BYU search engine (Davies, 2004), annotated with the CLAWS tagset (Fligelstone et al., 1997), while for the NKJP, we used the Poliqarp search engine (Przepriókowski et al., 2012) annotated with the Morfeusz tagset (Woliński, 2006). In both cases, we used regular expressions designed to locate verb-noun collocations that matched the specific query syntax of the given search engine (i.e., BNC vs. NKJP). The searches were lemmatic, meaning that they queried for all possible word forms. This was particularly important in the case of Polish, which has a very rich inflectional morphology. For both corpus analyses, the span of the search window relative to the potential collocate was set to six items and the searches used combinations of various word orders. These steps were taken to maximize the morpho-syntactic permutations that would be returned by our queries, e.g., the frequency and MI score (the strength of association between words; see section “What Are Multi-Word Expressions and How to Identify Them?”) for “give a presentation” would also be derived from “a presentation was given,” “giving a presentation in the morning,” “she gave several presentations,” etc.

Using this approach we created the three lists of stimuli taking four main steps. We first searched for English verb-noun collocations using a combination of two methods: the MI score (in the formulation of Stubbs, 1995) and n-grams, with false positives (e.g., machine annotation errors) vetted out by the trained linguists from within our team. We established which collocations are congruent and sufficiently represented in English (i.e., we chose those with MI scores above 2.0 and high n-gram frequency in the BNC). The 1553 English collocations identified in this way were then translated into Polish word for word to create calques. Following this, we queried for the Polish equivalents of the collocational calques in the NKJP in order to verify that they were not typical in Polish (i.e., that they had a low MI score and n-gram frequency). This is how the initial list of calques was obtained.

Next, using the “phraseological approach” (i.e., native speaker intuitions, Siyanova-Chanturia and Omidian, 2019, see section “What Are Multi-Word Expressions and How to Identify Them?”), we picked 261 collocational calques that we evaluated as plausible in contemporary Polish (e.g., we heard some Polish people use them in conversations, or we considered them as highly likely to be used due to their semantic similarity to Polish MWE). We checked that they could be turned into their correct Polish equivalents by replacing the verb (e.g., ^∗^*dać masaż*, “give a massage” – a calque vs. *robić masaż,* “^∗^make a massage” – a correct Polish collocation), resulting in a list of 183 phrases. Subsequently, we queried the NKJP to confirm high MI scores and n-gram frequency in the correct Polish collocations. To rule out co-incidental similarities between collocations in English and Polish, we also searched for analogs of the correct Polish collocations in the BNC and removed them from the list of well-formed Polish collocations. This is how the final list of 183 correct Polish collocations and collocational calques was created.

Then, to each pair of correct collocations and calques we added an absurd collocation by randomly assigning a verb to the noun (e.g., ^∗^*sprzątnąć masaż,* “^∗^clean a massage”). To confirm that the combinations were semantically meaningless or paradoxical, we performed appropriate searches in the NKJP and BNC, verifying that the combinations had negative MI scores and no representative n-gram combinations. This is how the list of absurd collocations was created. Finally, we also controlled for the corpus frequency of the verbs used in the verb-noun expressions. As a result we ended up with three lists of expressions in Polish, each containing 183 items:

• (1) Correct, well-formed Polish verb-noun collocations (MI according to NKJP *M* = 9.01, *SD* = 3.62; verb frequency *M* = 3.41, *SD* = 0.72), e.g., *robić masaż*, “^∗^make a massage”; *mieć sens*, “^∗^have sense”; *pojechać autobusem*, “go by bus”;
• (2) Collocational calques from English, where the English verb was replaced by a Polish translation equivalent (MI according to NKJP *M* = 0.41, *SD* = 1.96; verb frequency *M* = 3.80, *SD* = 0.88), e.g., ^∗^*dać masaż*, “give a massage”; ^∗^*robić sens*, “make sense”; ^∗^*wziąć autobus*, “take a bus”;
• (3) Absurd verb + noun expressions, where the verb did not collocate with the noun (verb frequency *M* = 3.31, *SD* = 0.67), e.g., ^∗^*sprzątnąć masaż*, “^∗^clean a massage”; ^∗^*zjadać sens* “, ^∗^eat sense”; ^∗^*kartkować autobus*, ^∗^browse the bus.

Finally, each of the 183 collocations was embedded in a carrier sentence approximately 10 words long (following Molinaro and Carreiras, 2010). The same sentence was used across all three conditions (well-formed, calque and absurd) – which gave a stimuli set consisting of 549 sentences (183 sentences ^∗^ 3 expression categories), for example:

• Fizjoterapeuta *zrobił masaż/^∗^dał masaż/^∗^sprzątnął masaż* kobiecie z uszkodzonym kręgosłupem.
• (The physiotherapist ^∗^*did a massage/gave a massage/^∗^cleaned a massage* to a woman with a spinal injury).

All the phrases and the carrier sentences are presented in Supplementary Appendix 1. These sentences were divided into three lists, each containing 61 correct collocations, 61 calques and 61 absurd expressions, each presented in a different carrier sentence.

#### Procedure

Experiment 1 was run as an online questionnaire in Google Forms. Participants were asked to judge whether the presented sentences were natural and acceptable in Polish. We used a 5-point Likert scale, where 1 meant “not natural at all” and 5 meant “perfectly natural in Polish.” Each participant assessed one randomly assigned sentence list, i.e., 183 sentences (containing 61 correct collocations, 61 calques and 61 absurd expressions).

#### Statistical Analysis

In the current analysis we decided to account for some *a priori* predictions using contrasts (planned comparisons). Specifically, we established a repeated contrast matrix that allowed us to compare directly between the acceptability of the correct MWEs vs. absurd expressions, and the correct MWEs vs. calques. The analysis was performed using linear mixed-effects models, as implemented in the lme4 package (version 1.1.21; Bates et al., 2015) in R (R Core Team, 2017) using participants and sentences as crossed random effects. In the model, the dependent variable was the Acceptability score (from 1 to 5) for each sentence. As fixed effects we included: Type of collocation (correct, calque or absurd) and Proficiency (self-rated). To the categorial predictor of Type of collocation, we applied a repeated contrast such that we compared Correct vs. Calque, and Correct vs. Absurd. The continuous predictor of Proficiency score was centered and scaled. We fitted the maximal model first (Barr et al., 2013) including the bobyqa optimizer, and in case of non-convergence or singularities we simplified it following recommendations outlines in Bates et al. (2018). The final model included the by-subject and by-sentence random intercept and correlated slope for Type of collocation. We considered as significant any fixed effect with an absolute *t*-value higher than 2.

### Results

The descriptive statistics for the behavioral study are presented in Table 1.

**TABLE 1:** Experiment 1: Descriptive statistics.

Experiment 1	Raw mean	SD	Predicted mean
Correct	4.58	0.73	4.58
Calque	2.42	1.38	2.41
Absurd	1.29	0.73	1.29

The analysis of the acceptability judgments showed a main effect of the comparison between Correct vs. Calques (*t* = −22.97), such that the calques were assessed as less acceptable than the correct collocations. There was also a main effect of the comparison between Correct vs. Absurd (*t* = −57.76), such that the absurd collocations were assessed as less acceptable than the correct ones (see Table 2). Importantly, no main effect of Proficiency or its interaction with collocation Type was observed.

**TABLE 2:** Experiment 1: Fixed and random effects for the LME model of the acceptability score.

	Estimate	Std. Error	*t*-value	By-participant SD	By-sentence SD
Intercept	4.58	0.05	86.06	0.21	0.47
Correct vs. Calque	−2.17	0.09	−22.67	0.38	0.87
Correct vs. Absurd	−3.29	0.06	−57.04	0.23	0.46
Proficiency (self-reported)	−0.01	0.04	−0.01	−	
(Correct vs. Calque) * Proficiency	−0.11	0.07	−1.58	−	
(Correct vs. Absurd) * Proficiency	−0.07	0.05	−1.42	−	

### Discussion

In Experiment 1 we tested whether Polish bilingual native speakers living in a Polish-speaking environment would judge collocations calqued from English as acceptable. We also explored whether individual variability in L2 proficiency would modulate the judgments. Experiment 1 was a behavioral study aiming to check the acceptability of the correct collocations, calques, and absurd collocations. We expected that the collocational calques (^∗^*wziąć autobus*) and absurd expressions would be judged by native speakers of Polish as less acceptable than the correct collocations (*pojechać autobusem*). In accordance with our expectations, Polish native speakers found the native correct MWE fully acceptable (mean 4.59 on a 5 point Likert scale, where 5 meant “perfectly natural in Polish”). They also found absurd collocations unacceptable (mean 1.28, where 1 meant “not natural at all”). Collocational calques were closer to the mid-point of the scale (mean 2.39), and the difference between the correct MWEs and the calques was significant. Overall, this means that Polish native speakers judged the calques from English as significantly less acceptable than the correct MWEs. Importantly, participants’ self-rated proficiency in English did not have any effect on the judgments of any of the collocation types. Thus next, we conducted Experiment 2 to find out whether there would be any differences in the processing of the three types of collocations by Polish speakers with the knowledge of English and whether their English proficiency would modulate the effects.

## Experiment 2 EEG Study

### Methods

#### Participants

Thirty new participants took part in the Experiment 2, all native speakers of Polish, aged 22–43 years (*M* = 25.47, *SD* = 4.36). They were recruited via a job–hunting internet portal and were paid for their participation. Their knowledge of English, ranged from intermediate to advanced (range 46.25–98.75; *M* = 70.86, *SD* = 11.48), as measured with LexTALE (Lemhöfer and Broersma, 2012). LexTALE scores are interpreted as follows: scores above 80 indicate an advanced to proficient L2 user (C1 and C2 level CEFR), scores in the range of 60–80 indicate an upper intermediate L2 user (B2 level) and scores below 59 indicate an intermediate level user (B1 level) and below (Lemhöfer and Broersma, 2012, p. 341). Thus, our participants’ proficiency ranged between that of a pre-intermediate and proficient L2 user. Thus, our sample was characterized by quite a large variability in L2 English proficiency, between pre-intermediate and advanced, which actually adequately reflects the population of educated Poles.

#### Materials

From the 183 carrier sentences created for the behavioral study, we chose 120 (see Supplementary Appendix 1), for the ERP experiment. First, we eliminated sentences with 11 calques that were assessed as acceptable in Experiment 1 (the scaled acceptability rating for the sentence with the calque was over 0.78). We eliminated two sentences where the calqued expression was repeated due to technical error and 50 sentences where the collocational calques used the common verbs “take,” “adopt,” or “make,” to avoid over-representation of expressions with those words. The sentences were divided into three lists, each containing 40 well-formed collocations, 40 calques, and 40 absurd expressions. Within each list the carrier sentences did not repeat.

#### Procedure

Participants were tested individually in a dimly-lit, sound-attenuated room. They sat approximately 60–70 cm from the screen. They were asked to read the 120 sentences for comprehension one by one (i.e., one of the lists described above), which were visually presented on the screen word by word. The stimuli were presented centrally on a 17″ CRT screen, in white letters on a dark-gray background. Each word appeared for 300 ms followed by a 300-ms blank screen and the sentence order was randomized. Participants had to answer Yes/No comprehension questions for 20% of the sentences containing the correct Polish collocation (see Supplementary Appendix 1). For example, following the sentence *Po imprezie musieliśmy posprzątać bałagan i wymienić szybę w kuchni* (After the party we had to clean up the mess and replace a glass pane in the kitchen) the question was *Czy impreza przebiegła spokojnie?* (Was the party peaceful?). Participants answered the questions by pressing the corresponding Yes/No button on the keyboard. The comprehension questions appeared randomly across the whole experiment.

#### Electrophysiological Recording

The electroencephalogram (EEG) was recorded at 256 Hz from 32 Ag/AgCl scalp electrodes positioned at the standard 10–20 locations, mounted in an elastic cap, using the Biosemi Active Two recording system. Electrodes were initially referenced online to the Common Mode Sense electrode located at the C1 electrode and re-referenced offline to the mean of the left and right mastoids. The horizontal and vertical electro-oculogram (EOG) was recorded bipolarly using electrodes placed below and above a participant’s right eye and at the outer canthus of each eye, respectively.

The EEG signal was offline filtered with a band-pass filter (0.1–25 Hz frequency range; low cutoff slope: 24 dB/oct; high cutoff slope: 12 dB/oct). Before analyzing the data, artifacts (such as eye movements) were manually removed using independent component analyses (ICA) (Jung et al., 2000; Delorme et al., 2007). Additionally, other artifacts were defined as events in which there was a difference of ± 100 μV in amplitude within less than 50 ms, or when the absolute amplitude exceeded ±100 μV. Trials with artifacts (2.99%) were rejected and recordings from electrodes with a high level of artifacts (>%) were interpolated using the average value of the group of nearest electrodes. We applied a baseline correction to the target nouns, creating epochs from −150 to 900 ms. The accepted EEG epochs were obtained for each participant, each sentence, and each electrode across all conditions (Types of collocation: Correct, Calque, Absurd). Because the baseline derived directly from the noun onset would be affected by the processing of the previously presented verb, we assumed a baseline correction that was neutral with respect to the experimental manipulation. That is, we applied a baseline correction using the average EEG activity in the 150 ms prior to verb onset, which was pre-verbal. Importantly, because the baseline time window was defined as the 150 ms directly preceding the verb, we had to take into account any ERP effects that could have arisen before the noun was presented. Thus, our analyses encompassed the mean amplitude of both the verb and the noun. Consequently, we averaged and analyzed epochs with respect to the onset presentation of the verb of −150 to 900 ms for the analysis of the verbs, and, because the noun was presented 500 after the onset of the verb, we extracted the epochs (−150–0) 500–1400 ms for the analysis of the nouns. That is, we extracted epochs from −150 to 900 with respect to the presentation of the target words [−150–900 in the case of verbs, and (−150–0) 500–1400 ms in the case of nouns]. Still, for the verbs in our collocations we did not have any specific predictions because the analysis at verb-level was purely exploratory.

Event-related potential extraction, averaging and cleaning were conducted with EEGlab (Delorme and Makeig, 2004) and ERPlab (Lopez-Calderon and Luck, 2014) MATLAB software toolboxes. We analyzed the P300 component for which we selected the 250–350 ms time window after word-onset on a cluster of centro-posterior electrodes: CP5, CP1, CP2, CP6, PO3, PZ, and PO4. We also analyzed the N400 component for which we selected the 350–450 ms time window after word-onset on a cluster of central electrodes: CZ, PZ, CP1, and CP2. The electrode clusters were chosen directly based on the literature on the N400 and P300 attesting to the scalp distribution of these components (Molinaro and Carreiras, 2010; Vespignani et al., 2010; Siyanova-Chanturia et al., 2017). We selected the Pz electrode for visualizing the effects because it was shared between the two clusters corresponding to each component (N400 and P300).

#### Statistical Analysis

In the present study we explored whether calques would be processed differently from the correct L1 collocations. Still, to “situate” the calques we needed to evidence the difference between the correct and absurd conditions. In the analysis we again used contrasts (planned comparisons) to account for an *a priori* set of predictions (see section “The Current Study”). Specifically, we established a repeated contrast matrix that allowed us to compare directly between the processing of Correct vs. Absurd, and Correct vs. Calque. The reason to select those contrasts was theoretically driven. First, because in the literature on MWEs and collocations the P300 is used to show template matching mechanisms for linguistic information that is uniquely predictable, we assumed that the P300 should distinguish between both Correct and Absurd, and Correct and Calque. Also, since predictable word combinations should lead to a reduced N400 component compared to unknown or incorrect collocations, for the N400 the contrast between Correct vs. Absurd provided us with a “safety check” (predictable vs. unknown) and allowed to situate Calque relative to the Correct.

The analyses were performed using linear mixed effects models, as implemented in the lme4 package (version 1.1.21; Bates et al., 2015) in R (R Core Team, 2017) using participants and sentences as crossed random effects. In the models for electrophysiological data the dependent variable was the voltage (microvolts) for the selected time window and electrodes. As fixed effects for all the models we included: Type of collocation (correct, calque or absurd) and Proficiency (English LexTALE score). To the categorial predictor of Type of collocation we applied a treatment contrast where the correct condition was the baseline using the default contrast setting contr.treatment in R. Thus, we compared Correct vs. Calque, and Correct vs. Absurd. The continuous predictor of Proficiency was centered and scaled. We fitted the maximal model first (Barr et al., 2013) including the bobyqa optimizer. We considered as significant any fixed effect with an absolute *t*-value higher than 2.

### Results

#### Verbs

The analysis of Verbs was purely exploratory. The descriptive statistics for Verbs in the P300 (250–350 ms), and N400 (350–450 ms) time-windows are presented in Tables 3, 4.

**TABLE 3:** Experiment 2: Descriptive statistics for the P300-Verbs.

	Raw mean	SD	Predicted mean
Correct	0.64	5.85	0.60
Calque	0.80	5.71	0.83
Absurd	0.61	5.74	0.57

**TABLE 4:** Experiment 2: Descriptive statistics for the N400-Verbs.

	Raw mean	SD	Predicted mean
Correct	−0.36	6.78	−0.38
Calque	−0.14	6.32	−0.08
Absurd	−0.66	6.66	−0.68

**FIGURE 1:**
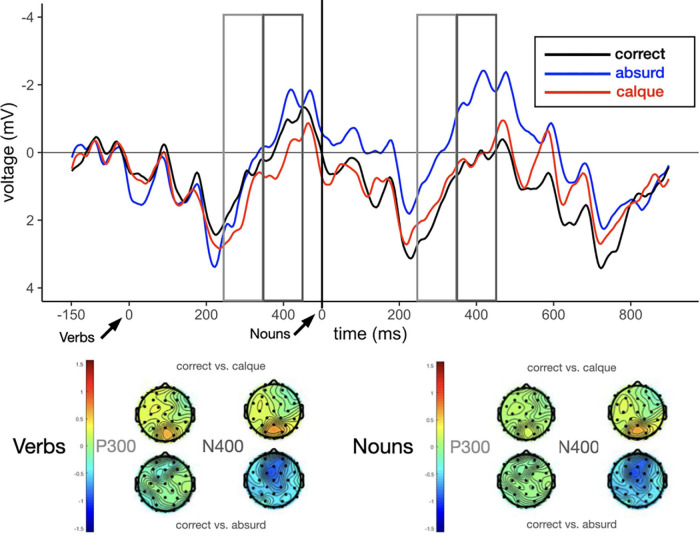
Average ERPs measured at the posterior (Pz) electrode site for the three types of collocations: correct (black), calque (red) and absurd (blue). The figure shows grand-averaged ERP waveforms for verbs and nouns, and the difference scalp maps in the P300 (250–350 ms) and N400 (350–450 ms) time window for the comparisons between correct vs. calque, and correct vs. absurd. Arrows indicate the verb and the noun onset.

The final models for Verbs in the P300 (250–350 ms), and N400 (350–450 ms) time-windows included the by-subject random intercepts and correlated slopes for Type of collocation. They also included the by-sentence intercepts and the correlated slopes for Type of collocation, Proficiency, and the interaction between Type of collocation and Proficiency. For Verbs, the analysis revealed no significant effect or interaction in any of the time-windows (P300 and N400), and no interaction with L2 Proficiency, as presented in Tables 5, 6, respectively. The grand-averaged ERP waveforms for Verbs are illustrated in Figure 1.

**TABLE 5:** Experiment 2: Fixed and random effects for the LME model of P300-Verbs.

	Estimate	Std. Error	*t*-value	By-participant SD	By-sentence SD
Intercept	0.60	0.27	2.27	1.08	1.88
Correct vs. Calque	0.23	0.34	0.68	1.47	2.31
Correct vs. Absurd	−0.03	0.26	−0.10	0.80	2.33
Proficiency (LexTALE)	−0.00	0.25	−0.00	−	1.68
(Correct vs. Calque) * Proficiency	0.08	0.35	0.24	−	2.39
(Correct vs. Absurd) * Proficiency	0.03	0.25	0.13	−	2.20

**TABLE 6:** Experiment 2: Fixed and random effects for the LME model of N400-Verbs.

	Estimate	Std. Error	*t*-value	By-participant SD	By-sentence SD
Intercept	−0.38	0.31	−1.23	1.20	2.35
Correct vs. Calque	0.30	0.42	0.71	1.85	2.68
Correct vs. Absurd	−0.30	0.40	−0.76	1.42	3.18
Proficiency (LexTALE)	−0.30	0.29	−1.07	−	1.92
(Correct vs. Calque) * Proficiency	0.36	0.42	0.86	−	2.64
(Correct vs. Absurd) * Proficiency	0.13	0.36	0.36	−	2.62

#### Nouns

The descriptive statistics for Nouns in the P300 (250–350 ms), and N400 (350–450 ms) time-windows are presented in Tables 7, 8.

**TABLE 7:** Experiment 2: Descriptive statistics for the P300-Nouns.

	Raw mean	SD	Predicted mean
Correct	0.92	6.86	0.88
Calque	0.62	7.16	0.64
Absurd	0.17	7.09	0.06

**TABLE 8:** Experiment 2: Descriptive statistics for the N400-Nouns.

	Raw mean	SD	Predicted mean
Correct	0.18	6.80	0.23
Calque	−0.33	7.03	−0.30
Absurd	−0.77	7.25	−0.75

Similarly to the Verbs, the final models for Nouns in the P300 and N400 time-windows included the by-subject random intercepts and correlated slopes for Type of collocation. They also included by-sentence intercepts and correlated slopes for Type of collocation, Proficiency, and the interaction between Type of collocation and Proficiency. The results for the respective time-windows (P300 and N400) will be discussed one by one below and presented in Tables 9, 10, respectively. The grand-averaged ERP waveforms for Nouns are illustrated in Figure 1.

**TABLE 9:** Experiment 2: Fixed and random effects for the LME model of P300-Nouns.

	Estimate	Std. Error	*t*-value	By-participant SD	By-sentence SD
Intercept	0.89	0.29	3.03	1.12	2.24
Correct vs. Calque	−0.24	0.37	−0.65	1.35	2.96
Correct vs. Absurd	−0.82	0.41	−1.99	1.58	3.15
Proficiency (LexTALE)	0.20	0.28	0.72	−	2.11
(Correct vs. Calque) * Proficiency	−0.01	0.40	−0.02	−	3.34
(Correct vs. Absurd) * Proficiency	0.19	0.40	0.46	−	2.98

**TABLE 10:** Experiment 2: Fixed and random effects for the LME model of N400-Nouns.

	Estimate	Std. Error	*t*-value	By-participant SD	By-sentence SD
Intercept	0.23	0.25	0.90	1.04	2.45
Correct vs. Calque	−0.53	0.34	−1.56	1.25	3.32
Correct vs. Absurd	−0.98	0.41	−2.40	1.75	3.67
Proficiency (Lextale)	0.14	0.24	0.57	−	2.24
(Correct vs. Calque) * Proficiency	0.03	0.35	0.07	−	3.33
(Correct vs. Absurd) * Proficiency	0.25	0.41	0.62	−	3.54

##### The P300 effect (250–350 ms)

In the P300 region, the analysis revealed a main effect of comparison between Correct vs. Absurd (*t* = −1.99) marginally significant, such that the Correct collocations yielded a more positive peak than the Absurd. No such effect was found between Correct and Calque, meaning that their waveforms were relatively similar (see Table 4, and Figure 1). The model did not find any main effect of Proficiency or its interaction with collocation Type.

##### The N400 effect (350–450 ms)

In this time window, the grand-averaged ERPs showed a distinct pattern based on Type of collocation. The analysis revealed a main effect of the comparison between Correct and Absurd (*t* = −2.89), such that for Correct collocations the negativity was reduced and for Absurd it was increased (see Table 10 and Figure 1). The comparison between Correct and Calque was not significant, meaning that their waveforms were relatively similar. Again, no effect of Proficiency or its interaction with collocation Type was found.

### Discussion

In Experiment 2 we tested whether Polish bilingual native speakers living in a Polish-speaking environment would read collocational calques from English differently from correct Polish collocations. We also explored whether individual variability in L2 proficiency would modulate the effects. Experiment 2 was an ERP study aiming to compare the processing of the correct collocations, calques, and absurd collocations. We expected that the nouns in the collocational calques (^∗^*wziąć autobus*) and absurd expressions would be less predictable for native speakers of Polish than the nouns in the correct collocations (*pojechać autobusem*). In accordance with our expectations, reading the nouns in the correct Polish collocations yielded increased P300 and decreased N400 effects relative to the absurd collocations. However, contrary to our expectations, the collocational calques from English yielded results comparable to the correct MWEs. Similarly to Experiment 1, participants’ proficiency in English did not modulate the processing of any of the collocation types.

## General Discussion

This study focused on one type of multi-word expressions (MWEs), namely verb-noun collocations (e.g., *take a picture*). Its goal was to explore whether Polish native speakers are sensitive to cross-linguistic influence (CLI) from their L2 English in comprehending collocations and whether their sensitivity is modulated by their L2 proficiency. Although collocations are common in all natural languages, their processing is still under-researched in comparison to other MWEs (especially idioms, Siyanova-Chanturia, 2013).

Here, we zoomed in on the bilinguals’ L1 rather than their L2, in contrast to many other studies on collocation processing by bilinguals and language learners, which examined native collocation patterns in the L2 use (e.g., Siyanova and Schmitt, 2008; Durrant and Doherty, 2010; Yamashita and Jiang, 2010; Wolter and Gyllstad, 2011, 2013; Szudarski and Conklin, 2014). We did so to examine the possibility of reverse transfer, that is CLI from the L2 to the L1 (Jarvis and Pavlenko, 2008). CLI from the L2 to the L1 is ubiquitous in users of two languages (see Cook, 2003, Cook, 2016) and, according to Schmid and Köpke (2017), it is not restricted to contexts of migrants making little use of their L1 and immersed in the L2. However, reverse CLI from the L2 to L1 has not been previously explored in the case of MWEs, and especially collocations.

We investigated how the L1 verb-noun collocational patterns are processed by native speakers of Polish living in the L1 environment, assuming that they might be influenced by CLI from English, a frequent source of borrowing into Polish. We also assumed that the degree of CLI from English might be modulated by Polish speakers’ level of English proficiency. To trace the reverse CLI from the L2 English in the L1 Polish, we asked whether English collocational calques are (1) acceptable to Poles and whether (2) they are processed similarly to Polish collocations by Polish speakers of varying English proficiency. To this end, we embedded three types of Polish verb-noun expressions into sentences: correct Polish collocations (e.g., *pojechać autobusem –* go by bus), collocational calques from English, where the verb was replaced by a Polish translation of the English verbs (e.g., *wziąć autobus –* take a bus) and absurd collocations (e.g., *zjeść autobus* – eat a bus). All collocations were identified using the frequency-based approach (MI score) and the phraseological approach (native speakers’ intuitions, see Siyanova-Chanturia and Omidian, 2019).

To answer the first research question, in a behavioral study we presented L1 Polish participants of varying L2 English proficiency with the experimental sentences and asked them to rate the acceptability of those. The results were quite straightforward: a group of Polish native speakers explicitly judged most calqued expressions as less natural than the correct Polish collocations. The comparison of the sentence acceptability between the three types of stimuli revealed that the collocational calques were less acceptable than the correct Polish collocations, but more so than the sentences containing absurd expressions. The result for calques stands in contrast to Laufer (2003), whereby bilingual speakers accepted collocational calques from their L2 as correct in their L1. However, in Laufer’s study the participants were immersed in the L2 environment, whereas those in our study where immersed in the L1. As such, our result suggests the collocational calques from English have not yet fully penetrated the Polish language, and Polish-English bilinguals living in the L1 environment are able to detect them as non-native in Polish. Interestingly, this effect was independent of participants’ self-rated English proficiency, so, contrary to our expectations, even participants who knew English better, were still able to detect the calques from English.

To answer the second research question and explore the neural response to the calques from L2 to L1, we ran an ERP experiment in which participants were asked to read sentences that included calques, correct collocations and absurd expressions. On the basis of the behavioral study and previous research (Molinaro and Carreiras, 2010; Vespignani et al., 2010; Siyanova-Chanturia et al., 2017), we expected that native speakers of Polish would show sensitivity to the anomaly in both calques and absurd collocations. This sensitivity would be manifested by the modulations of N400 and P300. For the correct Polish collocations, we expected to obtain a reduced N400 to the nouns, which would reflect the fact that once a comprehender starts processing an MWE, the subsequent words of the MWE become more predictable. We also assumed a more positive amplitude of the P300 component to the nouns in the correct collocation condition, which would reflect a mechanism of template matching for MWEs. Conversely, for both calques and absurd collocations we expected a more negative N400, and a less pronounced P300 component to the nouns, relative to correct collocations. For the verbs we did not have any explicit predictions.

For the verbs, we did not find any significant effects of comparisons between the correct collocations and calques, as well as the correct and absurd collocations, time-locked to the P300 and N400 regions. All the effects that were found pertained to the nouns. However, contrary to our expectations, none of the observed effects to the nouns described below were modulated by the participants’ L2 proficiency.

As expected, for the correct collocations, we indeed found an increased P300 amplitude for the correct collocations as compared with the absurd expressions and a reduced N400 to the nouns, which is in line the results from previous ERP research on MWE processing (Molinaro and Carreiras, 2010; Rommers et al., 2013; Vespignani et al., 2010; Siyanova-Chanturia et al., 2017). Following Siyanova-Chanturia et al. (2017), we interpreted the P300 for the correct collocations as typical of stronger template matching^2^. However, we do realize that the P300 results presented in our study are more supported by statistical models rather than by the waveforms (in comparison to the Pz electrode plots by Molinaro and Carreiras, 2010 or Siyanova-Chanturia et al., 2017). The reduced N400 amplitudes when processing correct Polish collocations (relative to the processing of calques and absurd collocations) provides evidence that native Polish collocations are processed with greater ease relative to other word combinations. In a given sentence context, the appearance of the verb belonging to an MWE made the noun that follows the verb in the MWE more predictable for Polish native speakers, possibly due to how the structure of collocations and their meaning are both stored in memory.

Although the ERP research on MWEs processing is still limited (Siyanova-Chanturia et al., 2017), the results presented here are partly in line with previous studies on MWEs, where the increased N400 was detected in response to novel, ill-formed, and metaphorical expressions (Molinaro and Carreiras, 2010; Vespignani et al., 2010). We found that absurd collocations elicited larger amplitudes of the N400 component than the correct collocations. However, in contrast to our expectations, we found there was a reduced amplitude of the N400 for nouns in the collocational calques, which mirrored the N400 to the correct collocations. In other words, the calques evoked similar neural responses to the correct collocations. This showed the participants’ lack of sensitivity to the anomaly included in the calque and an effect of semantic integration of the nouns in both the correct collocations and calques. The effect might be explained in two ways.

First, it might mean that due to the borrowing and assimilation mechanisms (see Otwinowska-Kasztelanic, 2000; Winford, 2003) the English calques have already become part of the Polish language, and are common enough to be stored in memory and processed just like correct Polish collocations (so even the people who do not know English will treat and comprehend those calques on par with the correct Polish MWEs). However, this is not reflected at the behavioral level where sensitivity to the calques was still observed. Secondly, and more plausibly, the results mean that CLI from English plays a role, such that Polish L2 users are sensitive to collocational patterns present in both their L1 and L2. The collocational calques, although still unacceptable in Polish, were congruent with the L2 English collocational patterns. The lack of significant differences between calques and the correct collocations at noun level in the P300 and N400 time windows might be a carry over effect of the congruency between the L2 collocational pattern and the calqued collocation in the L1. The effects of L1-L2 congruency were previously noted in studies on the processing of collocations in the L2 (e.g., Yamashita and Jiang, 2010; Wolter and Gyllstad, 2011, 2013; Szudarski and Conklin, 2014). Assuming that L1 knowledge influences the use and processing of the L2, the reverse pattern is also possible (Cook, 2003, Cook, 2016). The mechanism underlying the processing of the English calques in Polish might resemble the one proposed by Wolter and Gyllstad (2011, 2013). When the Polish user of English encountered a collocational calque from English, reading the verb in the collocation could trigger the activation of the collocation’s English translation equivalent. This would lead to facilitated access to the L2 collocation. No difference in the P300 and N400 effects between the correct collocations and calques could, thus, result from participants’ familiarity with calqued expression in their L2 English.

However, if calques are processed according to the L2 pattern due to CLI, as indicated by ERP data, then why do the behavioral results show speakers’ sensitivity to them? The discrepancy in the results might be due to the very nature of the two tasks we used: acceptability judgments vs. reading for comprehension. Those two types of tasks involve different types of processing, the former being more explicit than the latter (Carrol and Conklin, 2020). In the behavioral study, participants were asked to identify the collocation, carry out a lexical search, and analyze the words to detect whether such a word combination is acceptable or not. Moreover, this task did not include any time limits, and most likely engaged participants’ metalinguistic knowledge, thus increasing the chances of negative responses to calques that are “not quite right” in Polish. In contrast to the behavioral task, the ERP measurement did not rely on any acceptability decision and the task did not make participants explicitly focus on the colocations or consider their correctness in Polish. Participants had to read the sentences for comprehension (like in Molinaro and Carreiras, 2010; Vespignani et al., 2010; Rommers et al., 2013) and answer simple yes/no questions. In such a task, identifying the predicted word does not require active lexical search (for the argument see Vespignani et al., 2010). Thus, the system could accept the collocational calques as correct even though the verb did not quite match the native pattern. Still, in the case of nouns in the calques, enough semantic information was activated compared to the nouns in the correct collocations, which also resulted in reduced N400 components.

As a caveat, the system’s acceptance of collocational calques as correct may have stemmed from participants’ gradual exposure to the calques during the ERP experiment. In the Supplementary Material we present a series of **Additional Analyses** including the trial number and the interaction of the condition with the trial number for the nouns. In a nutshell, all the models showed all the main effects obtained previously and there were no effects of the trial. However, the models also demonstrated interactions between the trial and condition. The results were most informative for the N400 to the noun, revealing a stable pattern for the correct collocations and the absurd collocations, but a gradual shift for the calques, which were first processed more like the absurd collocations, and only later like the correct ones. We can speculate that the results reflected some dynamics in the system, such that with longer exposure to the claques participants activated their L2 semantic networks and the memory traces for the L2 collocational patterns were becoming more active. Thus, the participants in our study, gradually exposed to the calques, were more likely to process them as the correct collocations.

Yet another possibility explaining the ERP results for the collocational calques is that the verbs in the calques were less semantically constraining than the verbs in the correct collocations, which meant that they connected to other words more readily and were plausible in a wider subset of sentences. Thus, possibly the presence of the calqued verbs in the sentence context was plausible enough to pre-activate a wide range of nouns to follow. This might depend on how constraining the sentence context was and how plausible a given verb was in that context. Unfortunately, we did not control for cloze-probability, which is a limitation of this research. As demonstrated by Carrol and Conklin (2020), both cloze-probability and MI score could indicate how easily a collocation can be integrated into the sentence. Also, future studies should test participants’ collocational repertoire and consider it in the selection and creation of the materials.

## Conclusion

Due to the influence of the L2 on the L1, in some contexts, explicit judgments between native and non-native MWEs, and especially collocations, might become fuzzy (Laufer, 2003). Because evidence of this CLI phenomenon is scarce, we tested for the influence of L2 English, a prestigious global language, on the L1 Polish collocations of Poles living in an L1 environment. Although the behavioral results indicated that native speakers of Polish assessed the collocational calques from English as less acceptable than the well-formed Polish collocations, we did not find evidence that those two types of expressions differed considerably at early stages of processing (as evidenced by the P300 and N400 components), which preceded a conscious decision about the correctness of the sentences. This suggests a dissociation between explicit offline judgments and indices of online language processing measured by the ERPs. It might also indicate that the influence of English is so pervasive that Poles are becoming more and more oblivious to the collocational calques from this language. We believe that our study provides tentative evidence for this, and that calques from English have penetrated the Polish language to the extent that they are no longer detected as anomalies, at least at the initial stages of processing. We conclude that the neural response to the calques and the explicit judgments about them indicates different levels of language processing that are to some extent dissociable. Crucially, the fact that the effects reported in the current study were not modulated by L2 proficiency suggests that borrowing and CLI from English, whose influence is ubiquitous, may lead to the assimilation of L2 calques in Polish. Calqued MWE might soon become fully integrated with the Polish language system, leading to language change at the societal level.

## Data Availability Statement

The raw data supporting the conclusions of this article will be made available by the authors without undue reservation.

## Ethics Statement

The studies involving human participants were reviewed and approved by the Research Ethics Committee at the Institute of Psychology, Jagiellonian University, approval number KE/10/032018 from 5.05.2018. The patients/participants provided their written informed consent to participate in this study.

## Author Contributions

AO, MM, JD, JS, MO, and ZW: conceptualization. MM and MO: data curation. MM, AC, and MO: formal analysis. ZW: funding acquisition. MM: investigation. AO, MM, and JD: methodology. AO and MM: project administration and validation. MM, MO, and ZW: resources. MM and ZW: supervision. AC: visualization. AO, AC, and MO: writing – original draft. AO, MM, AC, JS, MO, and ZW: writing – review and editing. All authors contributed to the article and approved the submitted version.

## Conflict of Interest

The authors declare that the research was conducted in the absence of any commercial or financial relationships that could be construed as a potential conflict of interest.

## Publisher’s Note

All claims expressed in this article are solely those of the authors and do not necessarily represent those of their affiliated organizations, or those of the publisher, the editors and the reviewers. Any product that may be evaluated in this article, or claim that may be made by its manufacturer, is not guaranteed or endorsed by the publisher.

## Footnotes

1 The Mutual Information score is a measure of the strength of association between words x and y. It expresses the extent to which the observed frequency of co-occurrence differs from what is expected based on chance. In a given corpus, the MI is calculated on the basis of the number of times a pair of items was observed together, versus the number of times each of the items that form the pair were observed separately. MI is more likely to give high scores to fixed phrases whereas the t-score will yield significant collocates that occur relatively frequently. The t-score measures the confidence with which we can assert that there is an association (Harper, 2008). While the MI score is more sensitive to conventionally lexicalized collocations (e.g., *post graduate*) whose internal collocates are unlikely to occur as separate words within a particular corpus, the t-score, by taking corpus size into account, is more sensitive to situationally conditioned collocations (i.e., ones specific to a given corpus) such as “rely on” or “in the form of the,” which are not conventionalized, but may recur in a particular specialized corpus.

2 It needs to be noted, however, that whether the P300 and N400 indeed reflect different underling mechanism remains open for future research specifically targeted at this question. The template matching effectively implies the predictability of one word following another, and such predictability also lies at the core of the reduced N400. We thank reviewer 1 for bringing it up to our attention.
