# Supplementary material for: Does L2 Proficiency Impact L2-L1 Transfer While Reading L1 Collocations? Evidence From Behavioral and ERP Data
Source: Front Psychol. 2021 Sep 29;12:673761. doi: 10.3389/fpsyg.2021.673761 (PMC8513778; doi:10.3389/fpsyg.2021.673761)
Supplement: Supplementary file 2 [file Data_Sheet_1.pdf]

Additional analysis by trial for nouns in the collocations. Supplementary material

We carried out additional analyses by trial for the Nouns in the P300 and the N400 time windows. They are summarized below.

The model for the P300 to the nouns is presented in Table 1a and Figure 1a below.

Table 1a. Experiment 2: Fixed and random effects for the LME model of P300-Nouns. Analysis by trial number

	Estimate	Std. Error	df	t value	by-participant	by-item
(Intercept)	0.88	0.29	86.32	3.00	1.12	2.28
Correct vs. Calque	-0.26	0.37	95.44	-0.71	1.35	03.04
Correct vs. Absurd	-0.85	0.42	79.58	-2.05	1.63	3.31
Lextale	0.16	0.28	85.96	0.57		2.11
Trial	0.18	0.11	40.82	1.67	0.53	
Correct vs. Calque * Lextale	0.04	0.39	113.06	0.10		3.34
Correct vs. Absurd * Lextale	0.31	0.38	100.42	0.81		3.00
Correct vs. Calque * Trial	0.10	0.06	128633.1	1.77		
Correct vs. Absurd * Trial	-0.25	0.06	127368.3	-4.16		

The results for the **P300-nouns** revealed the previously shown main effect of Correct vs. Absurd, and an interaction between the trial order and Correct vs. Absurd. The interaction showed that while there was a rise in voltage with more trials for the calques and correct collocations, the absurd collocations maintained the same voltage all along.

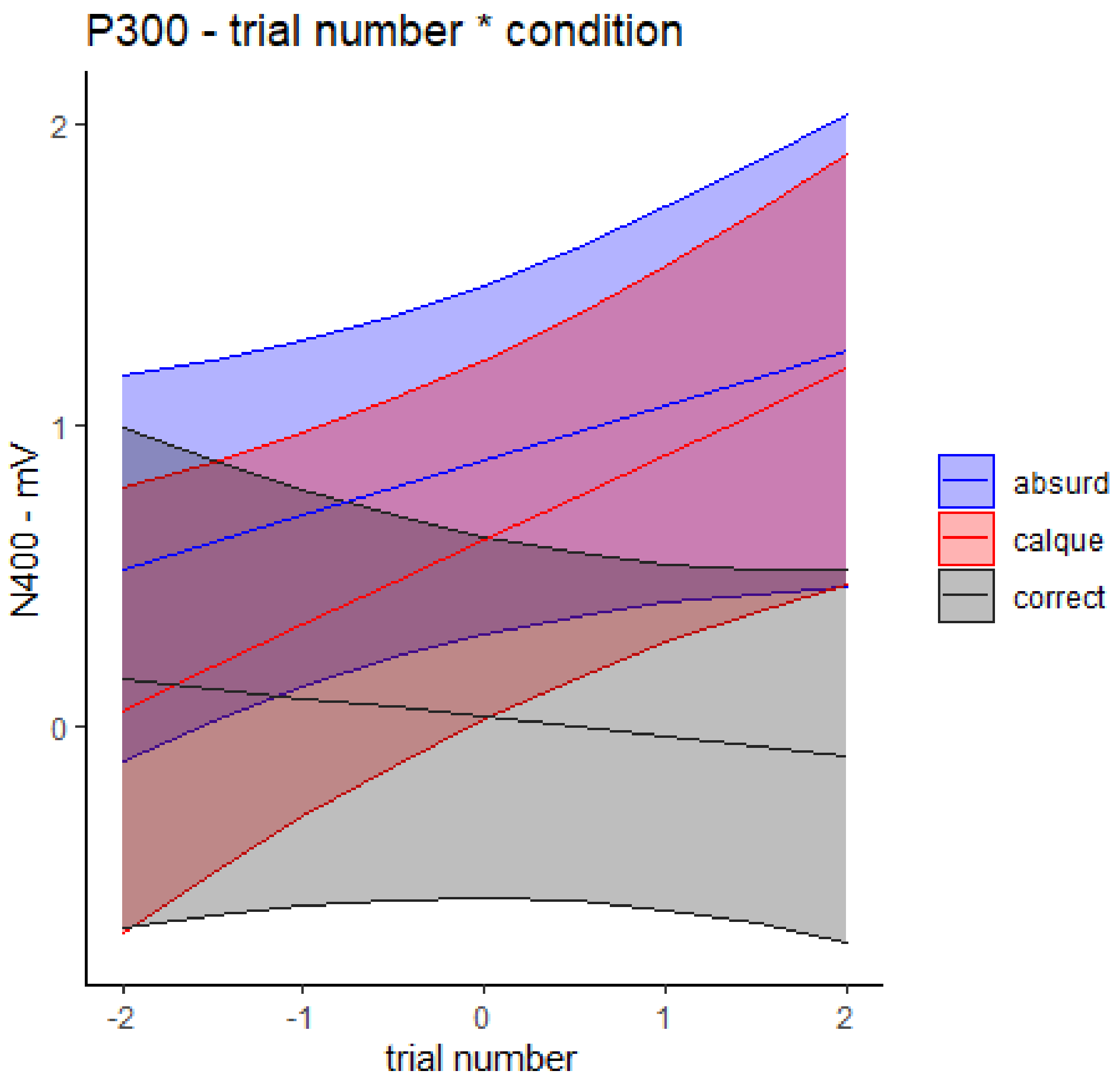

Figure 1a. Experiment 2. Interaction between the condition and the trial number representing changes in the voltage of the P300 component along the trials.

The model for the N400 to the nouns is presented in Table 2a and Figure 2a below.

Table 2a. Experiment 2: Fixed and random effects for the LME model of N400-Nouns. Analysis by trial number

	Estimate	Std. Error	df	t value	by-participant	by-item
(Intercept)	0.02	0.39	79.57	0.06	1.28	2.49
Correct vs. Calque	-0.23	0.41	87.95	-0.56	1.53	3.25
Correct vs. Absurd	-1.43	487	66.48	-2.94	02.03	3.36
Lextale	-0.16	0.31	80	-0.52		2.31
Trial	0.05	0.12	49.22	0.41	0.56	
Correct vs. Calque * Lextale	0.03	0.44	103.89	0.06		3.67
Correct vs. Absurd * Lextale	0.35	0.45	79.27	0.77		3.23
Correct vs. Calque * Trial	0.31	83	61267.47	3.74		
Correct vs. Absurd * Trial	-0.07	0.08	61662.62	-0.87		

The results for the **N400-nouns** showed the main effect of Correct vs. Absurd, and an interaction between the trial order and Correct vs. Calque. The interaction revealed that while correct and absurd maintained a stable voltage along the trials, the calques evoked less negative amplitudes with more trials, thus changing from the pattern of absurd to the pattern of correct collocations.

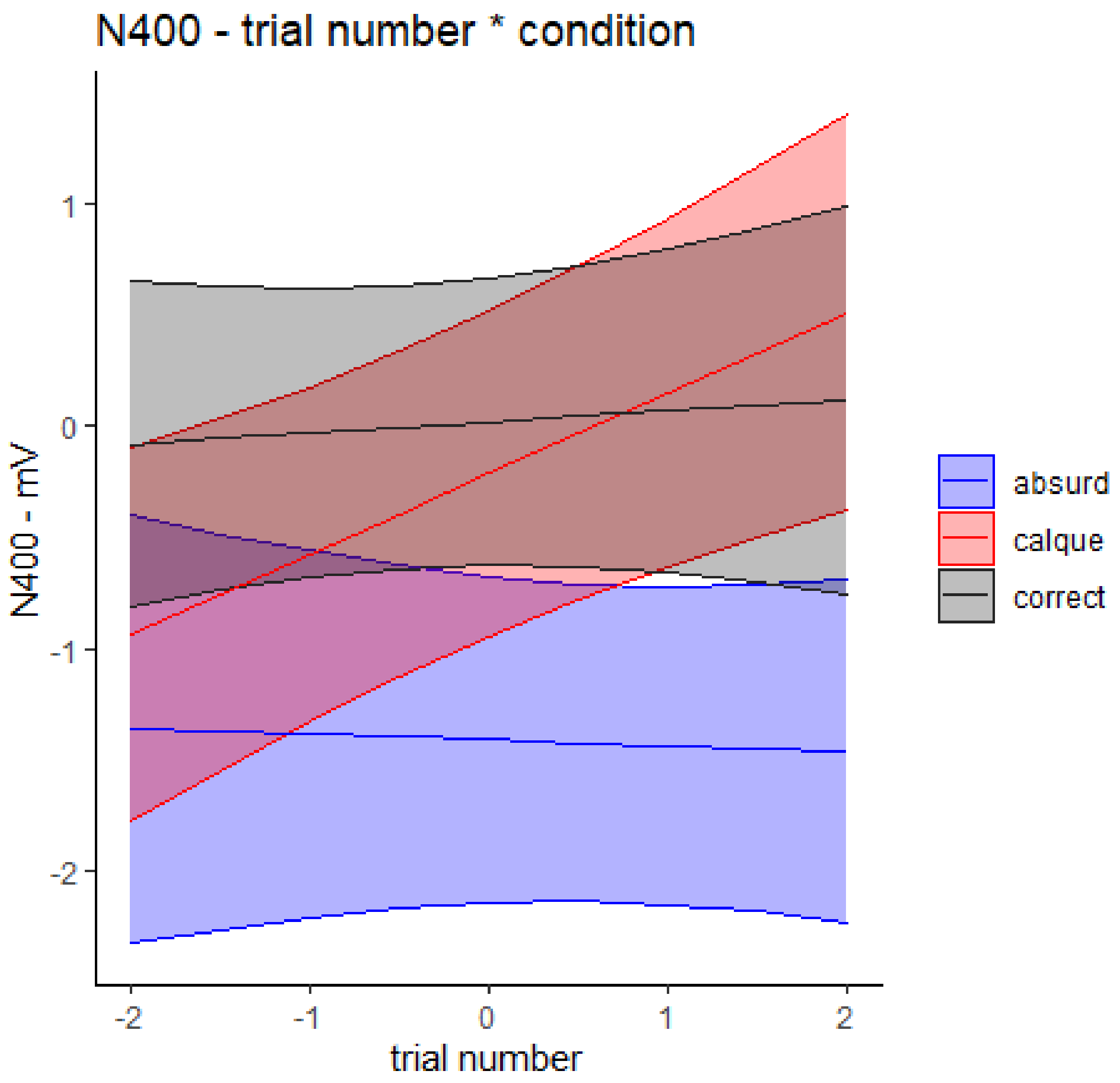

Figure 2a. Experiment 2. Interaction between the condition and the trial number representing changes in the voltage of the N400 component along the trials.
